# Cardiorespiratory endurance, muscular flexibility and strength in first-episode schizophrenia patients: use of a standardized fitness assessment

**DOI:** 10.1111/j.1751-7893.2011.00313.x

**Published:** 2011-12-13

**Authors:** Denise Gretchen-Doorly, Robin E. Kite, Kenneth L. Subotnik, Nicole R. Detore, Joseph Ventura, Andrew S. Kurtz, Keith H. Nuechterlein

**Affiliations:** UCLA Aftercare Research Program, Semel Institute for Neuroscience and Human Behavior, Los Angeles, CA, USA

**Keywords:** exercise test, health, measure, physical fitness, schizophrenia

## Abstract

**Aim:**

This study determined the fitness status and examined potential correlates of fitness in first-episode schizophrenia patients using a standardized fitness test protocol.

**Methods:**

A certified fitness instructor administered the Young Men’s Christian Association (YMCA) fitness test to 70 recent-onset schizophrenia participants within 3 months of entry into the study.

**Results:**

Percentile ranks of scores on muscular strength and endurance, muscular flexibility and cardiorespiratory fitness in our sample were all below the 50th percentile when compared with national norms in the United States. As expected, patients with a higher body mass index and those who smoked had poorer cardiorespiratory fitness. A non-significant trend indicated that patients with a longer duration of illness had worse cardiorespiratory fitness. Exposure to antipsychotic medication was unrelated to cardiorespiratory fitness.

**Conclusion:**

Results suggest that physical fitness is impaired and might decline over time in first-episode schizophrenia patients, but this needs to be confirmed in a longitudinal study.

## INTRODUCTION

Patients with schizophrenia have a 20% shorter life expectancy than the general population, and a greater vulnerability to several diseases, including diabetes, hypertension and coronary heart disease.^1–3^ Patients with first-episode psychosis are particularly vulnerable to adverse physical effects related to antipsychotic treatment, such as increased abdominal fat and metabolic disturbances.^4,5^ Recommendations for monitoring the health status of these patients are available in the United States, Europe and Australia.^6–8^ However, monitoring physical fitness in schizophrenia patients has not yet been included in standard health monitoring protocols.

Limited research on exercise and schizophrenia indicates that chronic patients spend less time engaged in regular physical activity than the general population.^9,10^ We could not locate any studies examining physical fitness in first-episode schizophrenia. Monitoring physical fitness in first-episode patients is particularly important, because increased rates of central obesity,^11^ the metabolic syndrome,^12^ glucose abnormalities^13–15^ and decreased insulin sensitivity^16^ can appear early in the illness.

The purpose of this study was to measure the three core components of physical fitness in a group of first-episode schizophrenia patients using the Young Men’s Christian Association’s (YMCA) fitness assessment protocol, which is a standardized procedure for adult physical fitness testing in the United States.^17^ We compared our fitness test results to the YMCA’s age- and gender-based normative data to determine if physical fitness in first-episode schizophrenia patients differed significantly from the general population of young adults in the United States. We also examined potential correlates of fitness capacity in young adult schizophrenia patients, including body mass index (BMI), duration of illness, exposure to antipsychotic medication and smoking status.

## METHODS

### Study site

The UCLA Aftercare Research Program is a longitudinal, NIMH-funded outpatient clinical research programme for first-episode schizophrenia patients. Patients are recruited from local inpatient and outpatient facilities in the Los Angeles area or are directly referred to the Program. Participants are provided with psychiatric medication management, psychoeducation, group skills training and individual case management for 18 months as participants in the Program.

### Sample

A total of 70 recent-onset schizophrenia patients from Sample 4 of the ongoing, longitudinal Developmental Processes in Schizophrenic Disorders project (K. H. Nuechterlein, PI) participated in the study. All patients had their first major psychotic episode occur within the last 2 years. The university’s institutional review board approved the protocol and all participants gave their written informed consent. The study conforms to the provisions of the Declaration of Helsinki, as revised in Tokyo 2004. All participants were medically cleared to participate in the fitness test.

Participants had a mean age of 21.8 years (SD = 3.7), a mean educational level of 12.3 years (SD = 1.8), a mean parental education level of 13.7 years (SD = 3.6) and a mean age of onset of illness of 20.7 years (SD = 3.8). The racial composition of the sample was 42% mixed race, 27% African American, 17% White, 13% Asian, and 1% American Indian or Alaskan Native. Thirty-nine percent of the sample was Hispanic and 76% was male. All patients were on oral risperidone awaiting random assignment to treatment with either oral or injectable risperidone as part of the research protocol.

### Measure

The YMCA Fitness Assessment protocol was developed by experts in physical fitness, exercise physiology, and sports medicine in the United States, and is one of the few fitness assessment protocols with an extensive normative database consisting of over 35 000 fitness tests.^17^ A certified fitness instructor administered the fitness test to participants within 3 months of entry into the Program (M = 4.2, SD = 3.6; median = 3.0; mode = 2.0 months).

The 3-minute step test, the modified sit-and-reach test, and the half sit-up test operationalized the variables of cardiorespiratory endurance, flexibility, and muscular strength and endurance, respectively.^17^ The test was given indoors to control for outdoor environmental conditions, such as temperature and humidity.

We used the Acuflex^®^ I (Novel Products, Inc., Rocton, IL, USA) sit-and-reach box to administer the modified sit-and-reach test. Participants sit on the floor with their back, hips and head against a wall, reaching forward three times. The third time the subject pushes the sliding device on the box as far forward as possible along the reach indicator and holds the final position for at least 2 s. The score for the test is the centimetres reached, recorded as the best of three trials.

The half sit-up test used a yoga mat outfitted with four 15-cm strips of textured tape placed 9 cm apart perpendicular to the body to ensure that participants raised their body to the required 30-degree angle. Subjects performed abdominal crunches by flexing their spine so that the fingertips of each hand reach the second strip of tape, then recline back so their shoulders touch the mat. The score for the test is the total number of properly executed repetitions at the end of 1 min.

The step test requires a participant to step up and down on a 30.5-cm high bench at the rate of 96 beats (24 steps) per minute for a total of 3 min. The test is scored as the recovery heart rate in beats per minute over 1 min, taken while seated beginning 5 s after the participant’s final step, with a lower heart rate representing better cardiovascular fitness. Heart rate was recorded using a digital heart rate monitor (Acumen^®^ EON Basix ES, Acumen Inc., Sterling, VA, USA).

## RESULTS

**FIGURE 1.**
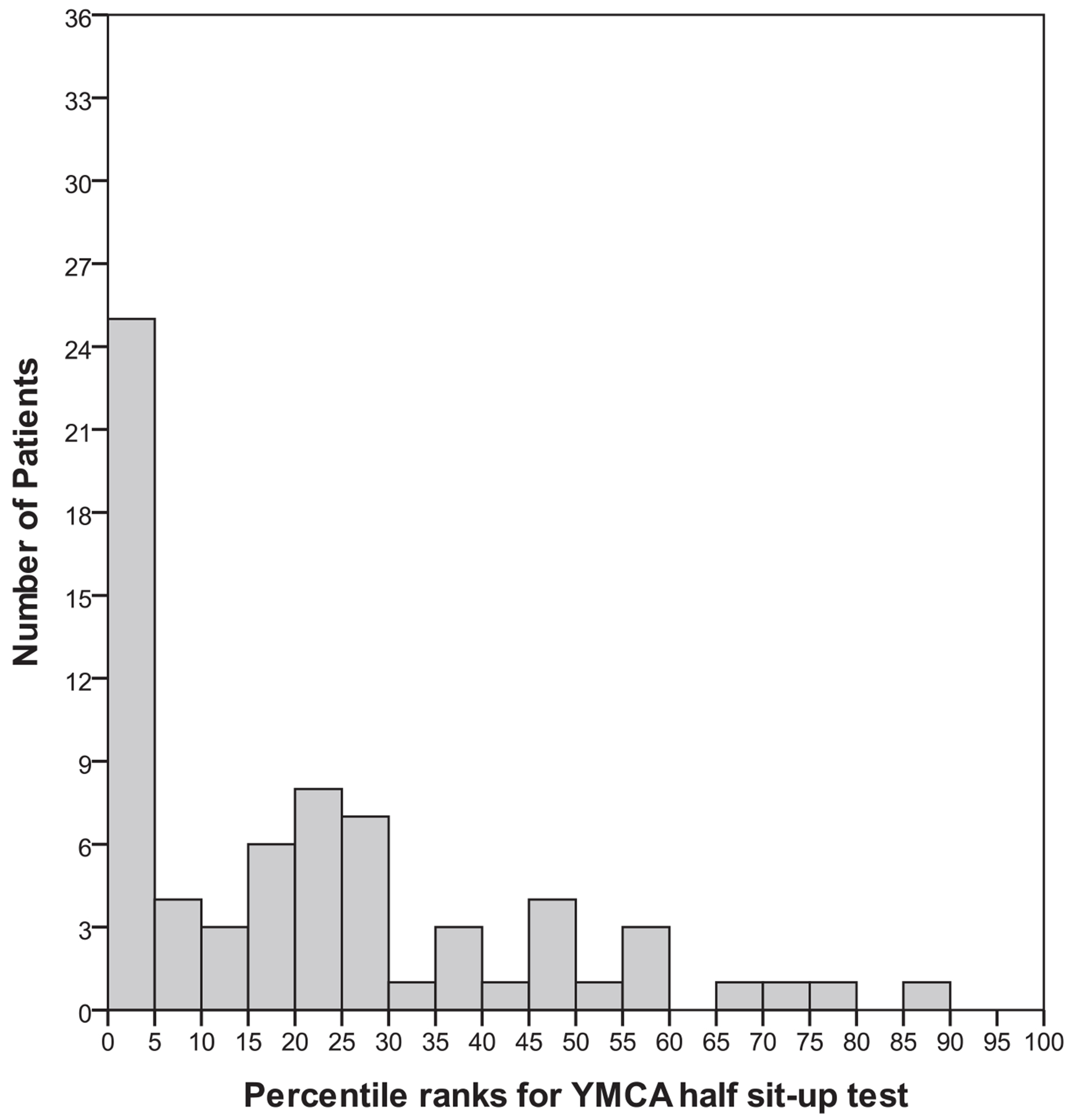
Percentile ranks of muscular strength and endurance from the Young Men’s Christian Association’s (YMCA) half sit-up test.

**FIGURE 2.**
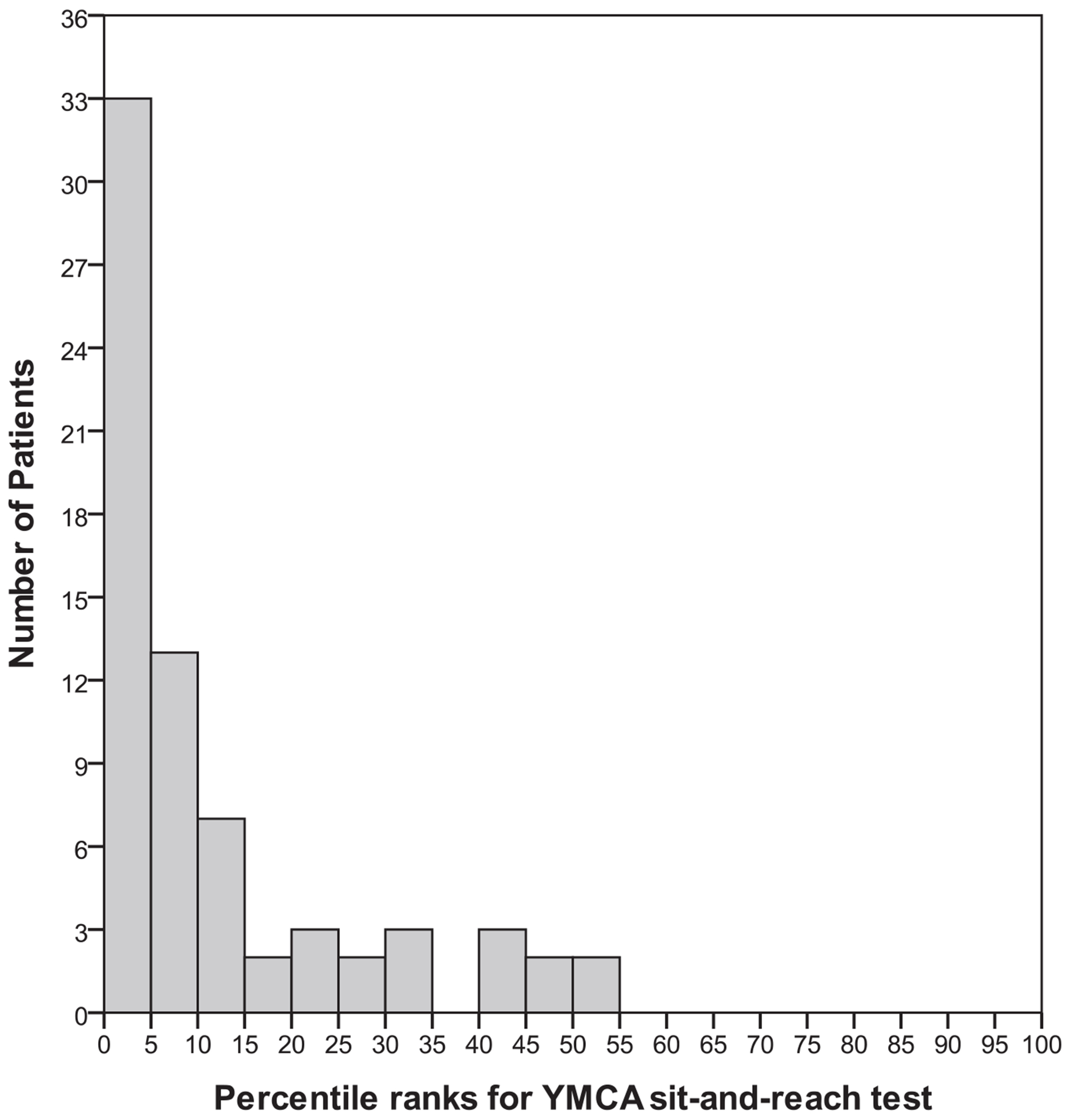
Percentile ranks of muscular flexibility from the Young Men’s Christian Association’s (YMCA) modified sit-and-reach test.

**FIGURE 3.**
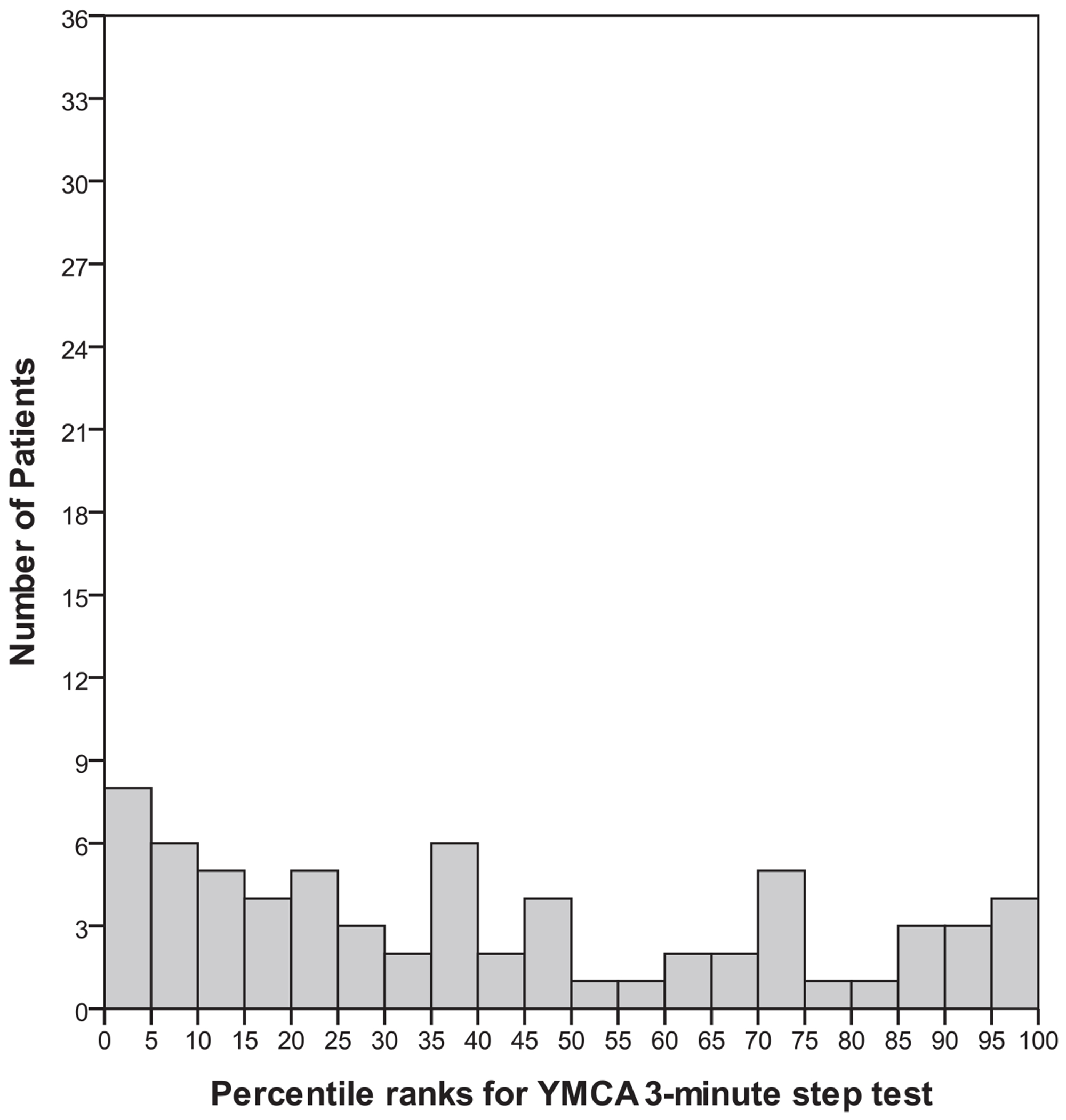
Percentile ranks of cardiorespiratory endurance from the Young Men’s Christian Association’s (YMCA) 3-min step test.

Fitness test raw scores were converted to age- and gender-based percentile ranks using normative data tables from the YMCA fitness test manual. Frequency histograms were generated to assess normality and are depicted in Figures 1–3. On average, the majority of patients scored near the 20th percentile for muscular strength and endurance (M = 20.8, SD = 21.3), the 11th percentile for muscular flexibility (M = 11.2, SD = 13.3) and the 40th percentile (M = 40.3, SD = 30.4) for cardiorespiratory fitness. The distribution of percentile rank scores for muscular strength and endurance (Fig. 1) and flexibility (Fig. 2) reflected a restricted range and positive skew and therefore were excluded from the correlational analyses. The distribution of percentile rank scores for cardiorespiratory endurance (Fig. 3) reflected a greater spread of scores, indicating more variability in the sample on this variable.

Baseline assessments indicated that the mean BMI in our sample was 28.2 (SD = 5.6) and that 50% were smokers. A BMI between 18.5 and 24.9 is considered normal, between 25.0 and 29.9 is overweight, and 30.0 or above is obese. Bivariate correlations indicated a significant relationship between low cardiorespiratory endurance and high BMI (*r* = −0.40, *P* = 0.001) and a non-significant trend for a relationship with increased duration of illness (*r* = −0.22; *P* = 0.099). A one-way analysis of variance revealed that smokers had somewhat poorer cardiorespiratory endurance scores than non-smokers (*F*(1,63) = 3.8; *P* = 0.057). Cardiorespiratory endurance was unrelated to medication exposure, defined as the number of days of prescribed medication, weighted by medication adherence (*r* = −0.05, *P* = 0.78).

## DISCUSSION

This study is one of the first to report on the physical fitness status of first-episode schizophrenia patients using a standardized fitness test protocol. Percentile ranks of scores on muscular strength and endurance, muscular flexibility and cardiorespiratory fitness in our patients were all below the 50th percentile when compared with national age- and gender-based norms in the United States. In addition, the distributions for muscular flexibility and strength and endurance had restricted ranges, with the majority of patients clustered at the low end of the scale. These results suggest that physical fitness is impaired in individuals with a recent first episode of schizophrenia.

We also examined potential correlates of cardiorespiratory fitness. As expected, patients with higher BMIs and those who smoked had poorer cardiorespiratory fitness. A non-significant trend indicated that patients who had a longer duration of illness had worse cardiorespiratory fitness. This suggests that physical health in the early course of schizophrenia might decline over time, but this possibility needs to be confirmed in a longitudinal study. It is noteworthy that exposure to antipsychotic medication was unrelated to cardiorespiratory fitness in our sample. This result mirrors other studies that document declines in the physical health in first-episode patients prior to detection of antipsychotic medication side effects.^4,5,11–16^ However, part of the reduced muscular flexibility in this group of patients might be due to antipsychotic side effects, even though muscular stiffness is less prevalent with second-generation antipsychotic medications.^18^

Given the observed impairments in physical fitness in our sample, targeted interventions deserve consideration. The health effects of regular physical activity are well established and include decreased mortality and morbidity related to cardiovascular disease and diabetes, as well as improvements in mental health, physical functioning and body weight.^19,20^ Specialized fitness programmes could represent an additional step toward the integration of physical and mental health care for individuals with severe mental illnesses, especially because preventative measures, like behavioural programmes, have been shown to be effective.^21–23^ Use of a standardized fitness assessment protocol, like the YMCA fitness test, can help researchers and clinicians measure the outcomes of programmes designed to engage this at-risk population in physical exercise.
